# Predictive value of hepatic transaminases during febrile phase as a predictor of a severe form of Dengue: analysis of adult Dengue patients from a tertiary care setting of Sri Lanka

**DOI:** 10.1186/s13104-021-05670-0

**Published:** 2021-06-30

**Authors:** Dickowita Kankanamge Dilani Priyangika, Gayani Premawansa, Madura Adikari, Sharmila Thillainathan, Sunil Premawansa, Bernard Deepal Wanniarachchi Jayamanne, Ranjan Premaratna

**Affiliations:** 1 University Medical Unit, Teaching Hospital, Ragama, Sri Lanka; 2 Colombo North Teaching Hospital, Ragama, Sri Lanka; 3 Zoology and Environmental Science, Faculty of Science, University of Colombo, Colombo, Sri Lanka; 4 Department of Public Health, Faculty of Medicine, University of Kelaniya, Kelaniya, Sri Lanka; 5 Department of Medicine, Faculty of Medicine, University of Kelaniya, Kelaniya, Sri Lanka

**Keywords:** Transaminases in Dengue, Dengue severity prediction, Dengue, And liver enzymes

## Abstract

**Objectives:**

Dengue viral infection is an ongoing epidemic in Sri Lanka, causing significant mortality and morbidity. A descriptive-analytical study was carried out using serologically confirmed Dengue patients during a 6 month period. The relationship between the elevation of hepatic enzymes and severity of Dengue was assessed after stratifying recorded maximum AST/ALT (SGOT/SGPT) values 2–15 times elevated and by the phases of the illness. Sensitivity, specificity, predictive values, and ROC curves were assessed using maximum values for AST and ALT.

**Results:**

Out of 255 patients, 107(42%) were females. The majority (52.9%) were in the 20–39 year age group. Only 19.6% had DHF. No statistically significant difference was noticed in the values of maximum transaminases during the febrile phase among DF and DHF patients. Higher sensitivity and low specificity with the 1–5 times elevation range was noticed, and a higher cut-off level of more than 5 times elevation showed low sensitivity and higher specificity. The combination of both transaminases cut-offs with age and sex also does not show clinically significant predictability of severe disease.

The AST and ALT elevations are not showing discriminatory predictive value on dengue severity. As different serotypes cause different epidemics, it is important to carry out large-scale specific studies considering the serotypes.

**Supplementary Information:**

The online version contains supplementary material available at 10.1186/s13104-021-05670-0.

## Introduction

Dengue Virus infection is known to cause significant morbidity and mortality in Sri Lanka. In 2017, total of 186,101 dengue cases were reported [1] with 440 deaths [1, 2]. It has been well reported in all districts of Sri Lanka and contributes to a substantial proportion of health costs [3]. The clinical entity of the disease can range from simple dengue fever, dengue haemorrhagic fever, and dengue shock syndrome.

Liver involvement in dengue infection is well described; however, evidence is limited whether the elevation of liver transaminases (AST—Aspartate transaminase or SGOT—Serum glutamic oxaloacetic transaminase and ALT—Alanine transaminase or SGPT—Serum glutamate-pyruvate transaminase) can be related to the severity of the disease. In 2009, the World Health Organization (WHO) revised its dengue guidelines and proposed severe organ impairment as one category of severe dengue in addition to severe plasma leakage and severe bleeding [4].

While some researchers show that elevated liver enzymes are a feature of severity, some other researchers concluded that it could occur at any point of the disease. Since there were limited studies carried out on this issue, the necessity of conducting a proper study has arisen to assess the relationship between elevated liver enzymes and the severity of the disease [5, 6].

If a relationship between elevations of liver transaminases and disease severity is elicited, liver transaminase elevation can be used as a predictor of disease severity, and those patients can be kept under close observation. Predicting the disease severity and prior preparedness and close monitoring will reduce future disease-related mortality.

### Ethical considerations and approval

Ethical approval was obtained by the Ethical Review Committee Faculty of Medicine, University of Kelaniya, Sri Lanka(P/75/04/2014). All participants gave informed written consent. Identifying information was kept entirely confidential.

### Study design and setting

A descriptive-analytical study was carried out at Colombo North Teaching Hospital (CNTH), Ragama, Sri Lanka. Data were collected for six months from 01st of May to 30th of October 2014.

### Inclusion criteria

All the patients who were confirmed using NS1dengue antigen and admitted to the two medical units were included in the study after obtaining informed written consent.

### Exclusion criteria

Patients with acute and chronic liver diseases were excluded from the study.

### Confirmation of diagnosis

Dengue was confirmed by clinical case definition along with Dengue NS1 antigen detection on acute serum samples. After considering serial clinical and laboratory data from the entire clinical course of the patient, treating clinicians categorised patients as having dengue fever (DF), Dengue haemorrhagic fever (DHF), or dengue shock syndrome (DSS) using WHO classifications 4, 6]. Dengue haemorrhagic fever required evidence of plasma leakage with or without other abnormal haematological parameters in addition to DF [4]. Dengue shock syndrome was defined when DHF was associated with either tachycardia or pulse pressure < 20 mmHg or systolic blood pressure < 90 mmHg [7].

### Data collection

Data were collected by using a questionnaire for patient interviews and a data extraction sheet for medical records.

### Data analysis

According to the laboratory standards, a level of 35 IU/l was taken as the upper limit of normal (ULN) for both AST and ALT. Subgroup analysis was carried out febrile phase subset using maximum AST and ALT (SGOT and SGPT) values after stratifying for three phases of dengue; febrile (days 1–3 of illness), critical (days 4–6), and convalescent (days 7–14) phases as defined by WHO 2009 [4]. Maximum AST and ALT values that occurred during the febrile phase form 2–15-fold rise (with ULN) were considered for analysis.

Statistical significance for continuous variables was determined using the Mann–Whitney U test. P value of 0.05 was taken as the significant cut off value. To figure out discriminatory performance, Receiver Operating Characteristic (ROC) curves were used in addition to screening statistics (Sensitivity, Specificity, Positive Predictive Value (PPV) and Negative Predictive Value (NPV).

Binary logistic regression also performed to assess the combined ability of two transaminases and combination with age and sex for the patients who showed either of maximum transaminases elevation for 3 categories (Normal, between 5 and tenfold rise and greater than tenfold rise) [8, 9] during the febrile phase of the disease. Data was analysed by SPSS (Version 22).

## Results

A total of 255 patients were recruited for the study. The majority were males (58%). Median age 27 years (Q1 = 21, Q3 = 40, IQR = 19). Dengue haemorrhagic fever was detected in fifty patients (19.6%). There were no cases of dengue shock syndrome. 14(5.5%) of all patients had bleeding manifestations, out of which 50% were DHF patients.

In the uncomplicated dengue fever category, males in the age group 20- 29 years were the highest prevalent group (19.2%) among the sample, while the least prevalent age-sex group was 60 years and above male group (1.6%) (Table 1). In the DHF group, females of the age 30–39 years had the highest prevalence (3.9%) while males of 60 years and above had the least prevalence (0.0%) (Table 1).

**Table 1:** Age categories of DF and DHF patients

Age category (years)	Males		Females		Total	
	DF (%)	DHF (%)	DF (%)	DHF (%)	DF (%)	DHF (%)
12–19	31 (12.6)	04 (1.6)	11 (04.3)	09 (3.5)	42 (16.5)	13 (5.1)
20–29	49 (19.2)	07 (2.8)	21 (08.2)	06 (2.4)	70 (27.4)	13 (5.1)
30–39	25 (09.8)	02 (0.8)	15 (05.9)	10 (3.9)	40 (15.7)	12 (4.7)
40–49	15 (05.9)	02 (0.8)	09 (03.5)	02 (0.8)	24 (09.4)	04 (1.6)
50–59	08 (03.1)	01 (0.4)	09 (03.5)	02 (0.8)	17 (06.7)	03 (1.2)
Above 59	04 (01.6)	00 (0.0)	08 (03.1)	05 (2.0)	12 (04.7)	05 (2.0)
Total	**132 (51.8)**	**16 (06.3)**	**73 (28.6)**	**34 (13.3)**	**205 (80.4)**	**50 (19.6)**

**Fig. 1:**
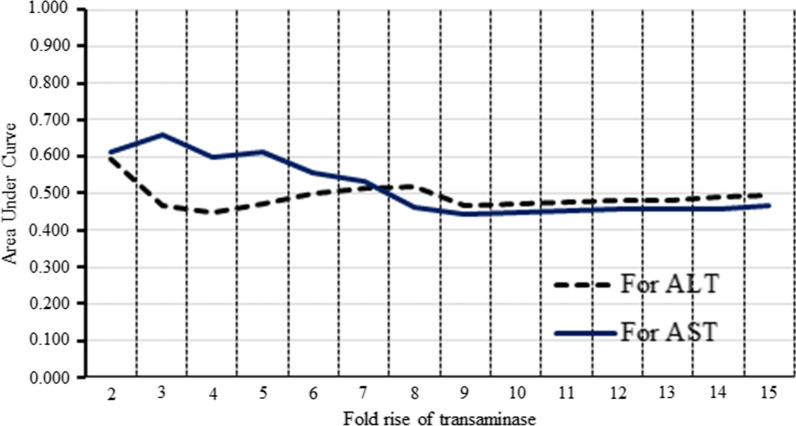
Area under the curve against fold rise of AST and ALT

Maximum ALT and AST values have occurred during the febrile phase among 59.2% (141/255) and 62.5% (159/255) patients, respectively (Additional file 1: Table S1). The subgroup of patients who showed a maximum rise of one or both transaminases during the febrile phase, comparing medians of both transaminases of DF and DHF patients, did not show a statistically significant difference. (Additional file 2: Table S2). Further analysis of the area under the curves (AUC) of ROCs for different cut-offs is shown in Fig. 1. All cut-off levels for both transaminases showed an area under the curve values within the range of 0.7–0.4 with a descending trend towards the high cut-off values (Fig. 1).

**Fig. 2:**
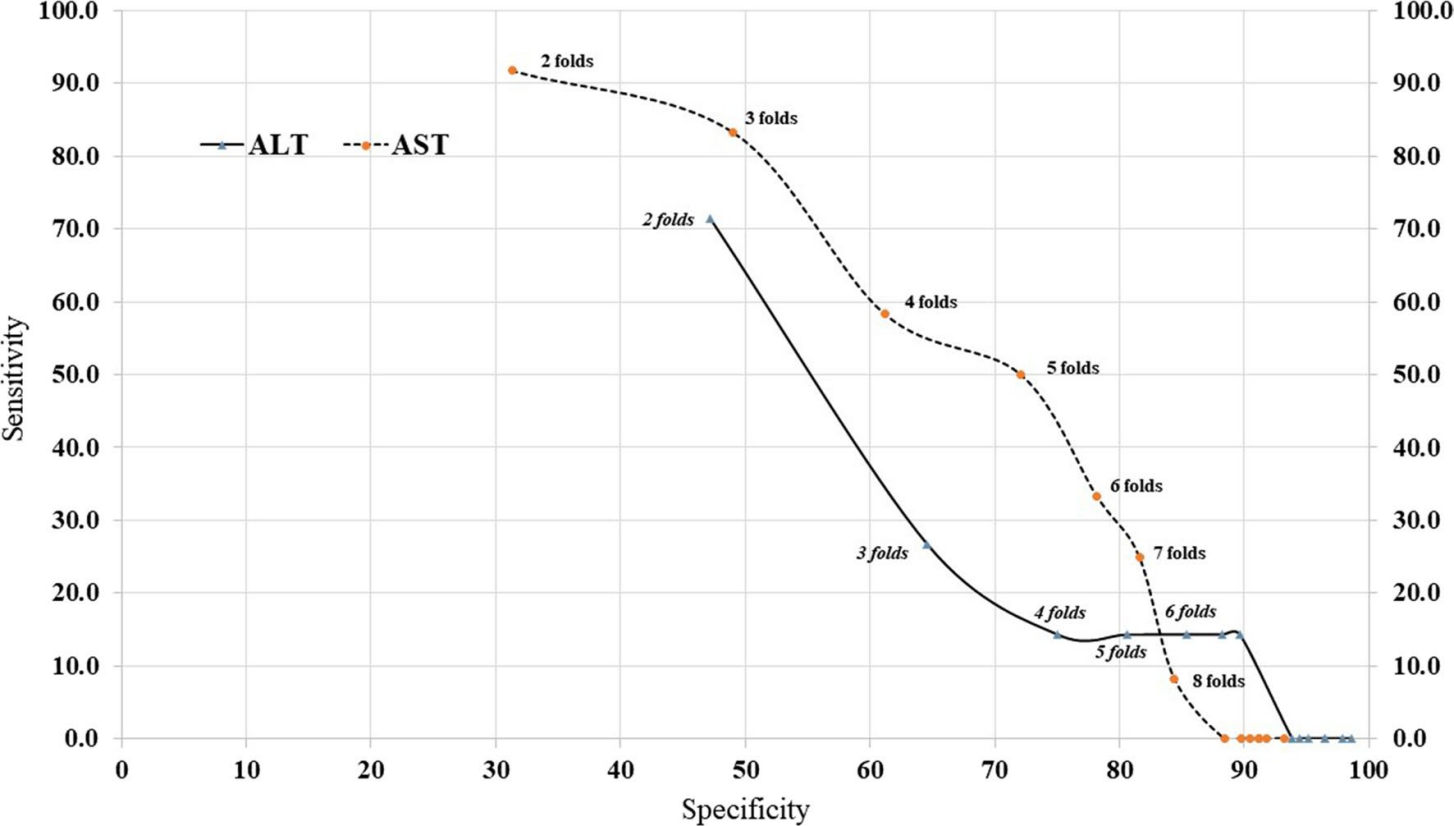
Specificity against Sensitivity for both transaminases all cutoff levels

Specificity vs sensitivity, plot shows AST lies at the specificity and accuracy triangle while ALT lies at the other half [10] (Fig. 2). All screening parameters showed the reach of their saturated value after an eightfold rise in both transaminases (Additional file 4: Figure S1 and Additional file 5: Figure S2).

Combined logistic regression models for the subgroup who had maximum elevation of transaminases during the febrile phase also showed statistically insignificant parameter estimates for both transaminases (AST p value = 0.712, ALT p value 0.701) at continuous scale alone. Adding age and sex did not improve the model (AST p value = 0.917, ALT p value 0.852). However, parameter estimate for sex had a significant predictive value for DHF in the model showing a significant Odds ratio (p value = 0.002) (Additional file 3: Table S3).

## Discussion

In this study, the maximum elevation of transaminases seen during the febrile phase was around 60% of the sample. Cut-off levels for both transaminases showed an area under the curve values within the range of 0.7–0.4 with a descending trend towards the high cut off values. Combined logistic regression models also showed statistically insignificant parameter estimates for both transaminases.

Acute liver is uncommon in dengue fever and was not reported in this study population. According to a study done by Trung and Kuo, only 1.1% had an acute liver failure with dengue infection [7, 11].

In the subgroup of patients who showed elevated transaminases during the febrile phase, a small proportion of DHF patients have shown the highest level during the febrile phase (Fig. 2). The median transaminases levels have shown no significant difference between DF and DHF patients (Additional file 3: Table S3). Liver function tests done at earlier dates might not reflect the extent of liver involvement in acute infection [12]. Analysis of statistics showed in Additional file 2: Table S2. So, there is no discriminatory value for median cut-off levels of transaminases.

According to ROC curve values, AUC between 0.9 and 1 considered as best predictors, 0.8–0.9 good predictors, 0.7–0.8 fair predictors, < 0.7 not good tests. Analysis of ROC curves using our data (Fig. 1), showed an area under the curve values below 0.7, which shows all the cut-offs fall into the "not good test” category.

There are only a few studies done and limited data available to find whether elevation of liver enzymes can be used as a predictor for DHF, including this study (Additional file 4: Figure S1 and Additional file 5: Figure S2). It was noted that results are varied, and many of the studies have shown that AST and ALT values cannot predict severity, while some studies have shown it has a certain degree of predictability as an independent predictor of DHF [13]. Some studies have shown that AST and ALT values may differ between DF and DHF [5–7, 14], few studies support AST or ALT as an independent predictor of DHF [10].

The exact reasons for elevation in liver enzymes in dengue fever are unclear. ALT is more specific for liver cell injury. Varying degrees of liver involvement is seen during acute dengue infection and are thought to result from hepatocyte apoptosis directly by the virus, hypoxic damage due to impaired liver perfusion resulting from fluid leakage, oxidative stress, or immune mediated injury [12]. On the other hand, elevation in AST can occur due to multiple pathologies involving the heart, muscle, red blood cells, kidneys, brain, and liver [15, 16].

## Conclusions and recommendations

In conclusion, the elevation of liver transaminases is common in dengue infection, however, use of elevated transaminases during the febrile phase alone is not a good predictor of the disease severity as a quick initial assessment. Combining it with one or more laboratory parameters like lowest total white cell count, lowest platelet count, etc. may be a good pathway for further evaluation and the timing of the febrile phase (day of fever). As different dengue virus serotypes cause different epidemics, it is also important to carry out further research in detail to explore the association of disease severity and elevated transaminases in that aspect as well with dengue patients.

This study was carried out among adults, and further studies in different age groups, including children, are suggested.

### Limitations

Since it is needed to find severity before the occurrence of critical phase, maximum AST/ALT value during the total disease course (occurred mostly during critical phase) cannot be used for clinical decision-making and predicting disease progression. Due to limited investigation facilities available in the public sector set up, it was not possible to assess co-infection with hepatitis viruses and hepatotoxicity. Patients who are known to have liver pathologies were excluded from the study during the time of selection. Also, screening for other virus infections was not routinely carried out due to the limited availability of the facilities.

## Supplementary Information

**Additional file 1: Table S1:** Maximum transaminases levels in different phases

**Additional file 2: Table S2:** Median comparisons (by Mann–Whitney U test)of the febrile phase maximum transaminases levels

**Additional file 3: Table S3**: Parameter estimates for binary logistic regression model for DF/DHF prediction from both transaminases, age and sex variables

**Additional file 4: Figure S1**: Screening statistics with fold rise of ALT

**Additional file 5: Figure S2**: Screening statistics with fold rise of AST

## Data Availability

The datasets generated during and/or analysed during the current study are not publicly available, but are available from the corresponding author on reasonable request.

## Abbreviations

ALT: Alanine transaminase
AST: Aspartate transaminase
AUC: Area under curve
CNTH: Colombo North Teaching Hospital
DF: Dengue fever
DHF: Dengue haemorrhagic fever
DSS: Dengue shock syndrome
NPV: Negative predictive value
PCR: Polymerase chain reaction
PPV: Positive predictive value
ROC: Respondent operative characteristics
SGOT: Serum glutamic oxaloacetic transaminase
SGPT: Serum glutamate-pyruvate transaminase
ULN: Upper limit of normal
WHO: World Health Organization
