# Supplementary material for: Predictive value of hepatic transaminases during febrile phase as a predictor of a severe form of Dengue: analysis of adult Dengue patients from a tertiary care setting of Sri Lanka
Source: BMC Res Notes. 2021 Jun 30;14:251. doi: 10.1186/s13104-021-05670-0 (PMC8243863; doi:10.1186/s13104-021-05670-0)
Supplement: Supplementary file 1 — Additional file 1: Table S1: Maximum transaminases levels in different phases [file 13104_2021_5670_MOESM1_ESM.docx]

**Table S1 :** Maximum transaminases levels in different phases

| **Phase** | **AST(%)** | **ALT(%)** |
| --- | --- | --- |
| **Febrile** | 159(62.4) | 151(59.2) |
| **Critical** | 34(13.3) | 39(15.3) |
| **Recovery** | 62(24.3) | 65(25.5) |
| **Total** | **255(100)** | **255(100)** |
