# Supplementary material for: Predictive value of hepatic transaminases during febrile phase as a predictor of a severe form of Dengue: analysis of adult Dengue patients from a tertiary care setting of Sri Lanka
Source: BMC Res Notes. 2021 Jun 30;14:251. doi: 10.1186/s13104-021-05670-0 (PMC8243863; doi:10.1186/s13104-021-05670-0)
Supplement: Supplementary file 2 — Additional file 2: Table S2: Median comparisons (by Mann–Whitney U test)of the febrile phase maximum transaminases levels [file 13104_2021_5670_MOESM2_ESM.docx]

**Table S2 :**  Median comparisons (by Mann-Whitney U test)of the febrile phase maximum transaminases levels

|  | **Maximum AST level**  **(U/L)** | | **Maximum ALT level**  **(U/L)** | |
| --- | --- | --- | --- | --- |
|  | **DF** | **DHF** | **DF** | **DHF** |
| **Mean** | 170.5 | 175.8 | 115.5 | 108.0 |
| **Median** | 109.0 | 178.0 | 76.0 | 87.0 |
| **Percentile 25** | 62.0 | 113.5 | 36.5 | 48.0 |
| **Percentile 75** | 187.0 | 243.5 | 139.0 | 126.0 |
| **Standard Deviation** | 190.6 | 77.9 | 121.0 | 82.8 |
| **Test statistic(Z)** | -1.715 | | -0.535 | |
| **p value** | 0.086 | | 0.592 | |
