# Supplementary material for: Predictive value of hepatic transaminases during febrile phase as a predictor of a severe form of Dengue: analysis of adult Dengue patients from a tertiary care setting of Sri Lanka
Source: BMC Res Notes. 2021 Jun 30;14:251. doi: 10.1186/s13104-021-05670-0 (PMC8243863; doi:10.1186/s13104-021-05670-0)
Supplement: Supplementary file 3 — Additional file 3: Table S3: Parameter estimates for binary logistic regression model for DF/DHF prediction from both transaminases, age and sex variables [file 13104_2021_5670_MOESM3_ESM.docx]

**Table S3** : Parameter estimates for binary logistic regression model for DF/DHF prediction from both transaminases, age and sex variables

| **Variable** | **Estimate** | **Std. error** | **p value** |
| --- | --- | --- | --- |
| Maximum AST Value* | 0.0004543 | 0.0043546 | 0.91690 |
| Maximum ALT Value* | 0.0013432 | 0.0072191 | 0.85240 |
| Sex (Males) | -2.5666132 | 0.8336310 | 0.002** |
| Age | -0.0298074 | 0.0227357 | 0.18984 |
| Constant | -0.8226872 | 0.7991965 | 0.30330 |

* During febrile phase, ** p value < 0.05
