# Supplementary figures and images for: Predictive value of hepatic transaminases during febrile phase as a predictor of a severe form of Dengue: analysis of adult Dengue patients from a tertiary care setting of Sri Lanka
Source: BMC Res Notes. 2021 Jun 30;14:251. doi: 10.1186/s13104-021-05670-0 (PMC8243863; doi:10.1186/s13104-021-05670-0)

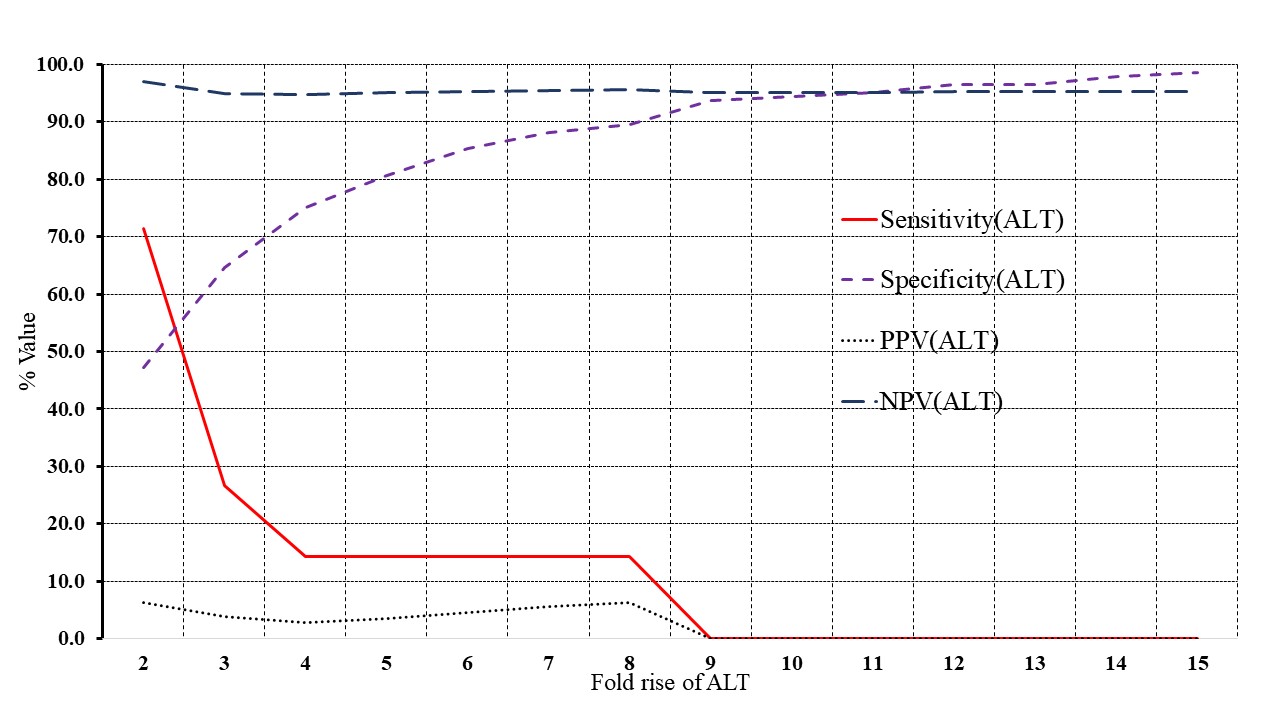

Supplement: Supplementary file 4 — Additional file 4: Figure S1: Screening statistics with fold rise of ALT [file 13104_2021_5670_MOESM4_ESM.jpg]

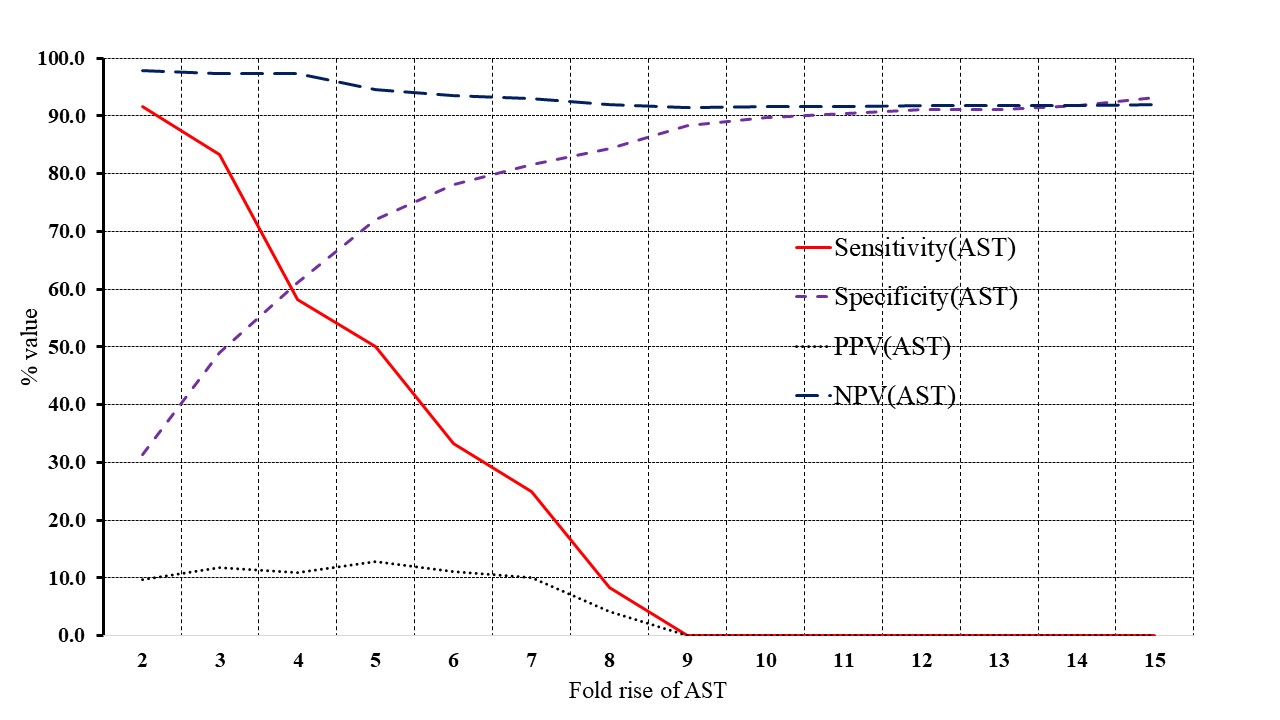

Supplement: Supplementary file 5 — Additional file 5: Figure S2: Screening statistics with fold rise of AST [file 13104_2021_5670_MOESM5_ESM.jpg]
